# Transcriptome Profile Analysis of Strawberry Leaves Reveals Flowering Regulation under Blue Light Treatment

**DOI:** 10.1155/2021/5572076

**Published:** 2021-06-12

**Authors:** Yuntian Ye, Yongqiang Liu, Xiaolong Li, Qing Chen, Yong Zhang, Ya Luo, Zejing Liu, Yan Wang, Yuanxiu Lin, Yunting Zhang, Xiaorong Wang, Haoru Tang

**Affiliations:** ^1^College of Horticulture, Sichuan Agricultural University, Chengdu 611130, China; ^2^Institute of Pomology and Olericulture, Sichuan Agricultural University, Chengdu 611130, China

## Abstract

Blue light is an important signal that regulates the flowering of strawberry plants. To reveal the mechanism of early flowering under blue light treatment at the transcriptional regulation level, seedlings of cultivated strawberry (*Fragaria × ananassa* Duch.) “Benihoppe” were subjected to a white light treatment (WL) and blue light treatment (BL) until their flowering. To detect the expression patterns of genes in response to BL, a transcriptome analysis was performed based on RNA-Seq. The results identified a total of 6875 differentially expressed genes (DEGs) that responded to BL, consisting of 3138 (45.64%) downregulated ones and 3737 (54.36%) upregulated ones. These DEGs were significantly enriched into 98 GO terms and 71 KEGG pathways based on gene function annotation. Among the DEGs, the expression levels of genes that might participate in light signaling (*PhyB*, *PIF*s, and *HY5*) and circadian rhythm (*FKF1*, *CCA1*, *LHY*, and *CO*) in plants were altered under BL. The BBX transcription factors which responded to BL were also identified. The result showed that the *FaBBX29*, one of strawberry's BBX family genes, may play an important role in flowering regulation. Our results provide a timely, comprehensive view and a reliable reference data resource for further study of flowering regulation under different light qualities.

## 1. Introduction

In the plant life cycle, the transition from vegetative to reproductive growth including flowering and consequent seed production is one of the most important developmental switches [1]. For crop production, flowering is a prerequisite for seeds and fruits that are later harvested [2]; hence, flowering time is considered a key agronomic trait in crop breeding works and research. The induction of flowering is regulated by multiple environmental cues, such as temperature, stress, and light [3, 4]. Plants continually monitor the intensity of light and its duration, spectrum, and direction so as to adjust their growth and development accordingly. Many plants rely on photoperiodic signals to control flowering's induction. Further, different light spectra can have different effects on the induction of flowering [5].

Much research into the photoperiodic flowering pathway has been carried out in plants. The external coincidence model was proposed in the twentieth century and supported by a known molecular mechanism of photoperiodic response in long-day plants, such as *Arabidopsis thaliana*, and in short-day plants, such as rice [6]. Numerous genes are involved in the molecular mechanisms that regulate the photoperiodic flowering pathway in *Arabidopsis*. The *AtFT* (FLOWERING LOCUS T) gene controls the flowering time by encoding a protein which is a key component of florigen in the companion cells of the phloem within leaves and which is transported to the shoot apex [7, 8]. The AtCO (CONSTANS) protein integrates the circadian rhythm and light signal inputs by governing the gene expression of *AtFT* in leaves by binding to the upstream promoter of the *AtFT* gene via a CCT (CONSTANS, CONSTANS-like, and TOC1) conserved domain [9, 10]. The abundance of AtCO protein is restricted by gene expression regulation as well as protein stability regulation. The AtCDF1 (CYCLING DOF FACTOR 1), a member of the CDF family, functions as a repressor of *AtCO* gene expression during the morning by binding to the CDF binding sites located near the transcription start site. Other members of the CDF family can also repress the *AtCO* and *AtFT* expression redundantly and delay the flowering time of *Arabidopsis* [11, 12]. Furthermore, the gene expression level of *AtCDF1* is regulated by the circadian clock core components. In the morning, AtCCA1 (Circadian Clock Associated 1) and AtLHY (LATE ELONGATED HYPOCOTYL) promote the expression of *AtCDF*, whereas PRR (PSEUDORESPONSE REGULATOR), another circadian clock component protein, represses transcription of *AtCDF* in the long-day afternoon [13–15]. In *Arabidopsis*, both AtFKF1 (FLAVIN-BINDING, KELCH REPEAT, F-BOX 1) and AtGI (GIGANTEA) form a complex in a blue light-dependent manner which mediates the ubiquitin-dependent degradation of the AtCDF1 protein. The function of AtFKF1 depends on the interaction between AtGI and AtCDF1 [16, 17]. The AtFKF1 also interacts with AtCO through its LOV domain and stabilizes the AtCO protein in the afternoon under a long-day condition [18]. Further, AtGI also interacts with the AtFKF1 homologs AtZTL (ZEITLUPE) and AtLKP2 (LOV KELCH PROTEIN 2) and synergistically degrades CDF2 protein together with AtFKF1. Additionally, it is known that AtGI can stabilize AtFKF1 and AtZTL [17]. The AtCOP1 (CONSTITUTIVE PHOTOMORPHOGENIC 1) protein, an E3 ubiquitin ligase, is involved in both the plant circadian clock and flowering time control as a key regulator in the light signaling pathway. The AtCO protein is reportedly degraded by a protein complex formed by AtCOP1, AtSPA1 (SUPPRESSOR OF PHYA), AtSPA3, and AtSPA4 during the night under both long-day and short-day conditions [19]. Recent research demonstrated that AtFKF1 acts as an upstream negative regulator of AtCOP1; specifically, AtFKF1 regulates AtCO stability and photoperiodic flowering through interaction with AtCOP1 and reduces the activity of AtCOP1 in a day-length-dependent manner [20].

There are distinct sets of photoreceptors in plants for sensing different light spectra, ranging from near-UVB (280–315 nm) to far-red (~750 nm) wavelengths. Plant flowering is promoted or delayed by different light qualities [21, 22]. A blue light treatment will promote floral initiation in *Arabidopsis* involving photoreceptors and critical genes of the light signaling pathway, and in plants Cryptochromes (CRYs) are blue light receptor capable of binding a FAD chromophore [23]. In an early study on *Arabidopsis*, the mutant plants (*cry2*) of *AtCRY2* flowered later than wild-type plants [24]. In *Arabidopsis*, AtCRY2 has been shown to activate *AtFT* expression in response to blue light by suppressing the degradation of AtCO protein. Under blue light, AtCRY2 interacts with the AtCOP1-AtSPA complex; this interaction further suppresses the COP1-dependent proteolysis of AtCO in the flowering pathway [25, 26]. Recently, the role of AtCIB (CRY2-interacting bHLH) proteins in mediating AtCRY2 inducing flowering pathway has been clarified. The AtCIBs are specifically involved in the AtCRY2 signaling pathway, for which the expression of corresponding *AtCIBs* is regulated specifically by blue light. For example, AtCIB1 stimulates *AtFT* expression by interacting with the chromatin DNA of the *AtFT* gene [27]. The AtCRY1, another CRY in *Arabidopsis*, also interacts with the AtCOP1-AtSPA complex and enhances the AtCRY2-AtCOP1-AtSPA interaction that contributes to flowering regulation [25]. In *Arabidopsis*, AtFKF1 protein plays a role as a blue light receptor by having the LOV (light, oxygen, or voltage, a subfamily of PAS domains) domain for the blue light perception, in that AtFKF1 interacts with AtGI to form a complex by absorbing blue light through its LOV domain. The AtFKF1-AtGI complex controls the expression of *AtCO* by degrading AtCDF protein via the formation of an AtFKF1-AtGI-AtCDF1 complex acting on the promoter of the *AtCO* gene [28, 29]. More recent research has indicated that AtFKF1 is able to control a robust *AtFT* mRNA induction through multiple feed-forward mechanisms. By interacting with AtCO, AtFKF1 stabilizes AtCO and blue light strengthens this interaction. Simultaneously, the complex of AtFKF1-AtGI removes the AtCDF1 protein on the promoter of the *AtFT* gene [18]. In *Arabidopsis*, AtFKF1 activated by blue light can interact with AtCOP1 and attenuate homodimerization of AtCOP1, to further control flowering time [20].

Strawberry is not only an important fruit crop but also a model plant in the Rosaceae family. Flowering of strawberry is a crucial trait of breeding, one that is affected by genetic background and various environmental factors [5, 30–33]. Blue light irradiation has been shown to affect many physiological aspects of strawberry, such as anthocyanin accumulation in fruits, induction of flowering, and *in vitro* growth of plantlets [32, 34, 35]. In a recent study of woodland strawberry (*Fragaria vesca*), in which seedlings were subjected to different light quality treatments, the results showed that *FvFT1* was involved in flowering induction; *FvFT1* is strongly activated by FR light yet weakly activated by blue light, and *FvFT1* mediated the promotion of flowering under blue light and FR light treatments in the perpetual flowering accession “Hawaii-4” [5]. However, the molecular mechanism by which the induction of flowering is regulated by differential light quality in cultivated strawberry (*Fragaria × ananassa* Duch.) remains unknown and awaits elucidation.

Given that previous studies have already showed that blue light promotes strawberry flowering via altered gene expression [5, 36], an overview that is aimed at revealing flowering induction under a blue light treatment at the transcriptome level is necessary. In the present study, we explored the effect of blue light (BL) and white light (WL) on flowering induction in cultivated strawberry plants. To do this, a transcriptome profile for strawberry leaves sampled from seedlings under different light quality treatments was determined using RNA-Seq technology. The BBX transcription factors responding to blue light were then identified. The results indicate that *FaBBX29*, a BBX family gene in strawberry, may play an important role in flowering regulation. Taken together, our results provide a comprehensive view of transcriptional regulation of flowering under the blue light treatment. This work also can serve as a reliable reference data resource in future studies of flowering regulation under blue light.

## 2. Materials and Methods

### 2.1. Plant Materials and RNA Extraction

Cultivated strawberry (*Fragaria × ananassa* Duch. “Benihoppe”) seedlings were grown in plastic pots (each 10 cm × 10 cm) containing a mixture of peat soil, coconut husk, and perlite in a ratio of 3 : 3 : 1 (*v*/*v*/*v*). Routine management practices were carried out in a greenhouse at Sichuan Agriculture University, in August 2018. For the light quality treatments, strawberry seedlings of uniform growth were divided into two groups (blue light vs. white light). These seedlings were subsequently subjected to light quality treatments in a growth chamber under controlled conditions (13 h dark photoperiod, at 15°C, 75% relative humidity) according to local meteorological information. Light-emitting diodes (LED) in blue (450 nm) and white (control) were affixed atop the chamber to provide the necessary irradiation (125 *μ*mol × m^−2^ × s^−1^) for plant growth. The seedlings' leaves were sampled at a time point when all seedlings had flowered under a given light quality treatment. The experiment was repeated three times resulting in samples for three replicates (at least 10 leaves per sample). All harvested samples were immediately frozen in liquid nitrogen and stored at –80°C for the downstream analysis.

From each sample, its total RNA was isolated using the modified CTAB (cetyltrimethylammonium bromide) method, as described by Chen et al. [37]. The integrity of hre RNA was evaluated by electrophoresis on 1% agarose gel, and RNA NanoDrop 2000 was used to measure the quantity of RNA.

### 2.2. Flowering Time Measurements and Statistical Analysis

Observations of flowering time were carried out from the beginning of each treatment. The flowering time of every seedling was recorded as the number of days elapsed since the treatment began to the first bloom [5]. These flowering time data were visualized in R software (v3.6.3).

### 2.3. The cDNA Library Preparation and Illumina Sequencing

After the total RNA extractions, the cDNA library preparation and Illumina sequencing were carried out by the Annoroad Gene Technology Corporation (Beijing, China). To generate the cDNA library, the NEBNext® Ultra™ RNA Library Prep Kit for Illumina® (#E7530L, NEB, USA) was used by following the manufacturer's recommendations, with index codes added to attribute the sequences uniquely to each sample. The mRNA was purified from total RNA by using poly-T oligo-attached magnetic beads, after which fragmentation was carried out using divalent cations under elevated temperature in a NEBNext First Strand Synthesis Reaction Buffer (5x). First strand cDNA was synthesized using a random hexamer primer and RNase H; the second strand cDNA synthesis was then done using a buffer, dNTPs, DNA polymerase I, and RNase H. Next, the library fragments were purified with a QIAquick PCR kit and eluted with EB buffer, and these underwent terminal repair, A-tailing, with an adapter added. The targeted products were retrieved and the PCR amplification performed, at which point the library was then complete. Finally, these cDNA libraries were sequenced on the Illumina Hi-Seq X platform and 150 bp paired-end reads were generated. Corresponding data pairs are indicated by the suffixes “_R1” and “_R2”; these applied to distinguish data generated from different ends of the same given cDNA library.

### 2.4. RNA-Seq Data Filtering and Genome-Guide Read Mapping

To obtain high-quality read data for the downstream analysis, the raw data from the sequencing platform were filtered by Trimmomatic software (v0.32) [38], to remove adapter sequence and low-quality reads resulting in a clean data set. At the same time, the Q30 contents of raw data and clean data were also calculated. Because the high-quality whole-genome sequence data of cultivated strawberry were recently published [39], a genome-guide assembly based on cultivated strawberry genome data was performed using the HISAT2-Stringtie pipeline [40]. The clean data were subsequently mapped back to the reference genome. Both the mapping rate and the expression level of transcripts were normalized by the TPM (Transcripts Per Kilobase Million) method. The analysis pipeline of HISAT2 and Stringtie was executed under their default program parameter settings.

### 2.5. Differential Expression Analysis

The R package “DESeq2” is a statistical routine for conducting the differential expression analysis of digital gene expression data, using a model based on the negative binomial distribution [41]. For this differential expression analysis with DESeq2, a matrix of read counts mapped to transcripts was generated from the Stringtie output data by using a python script (https://github.com/gpertea/stringtie/blob/master/prepDE.py) [40]. The results from DESeq2 include the adjusted *P* values of genes according to the BH approach; this applied for robust multiple hypothesis testing by controlling the false discovery rate (FDR). Those transcripts with a *P*adj < 0.05 and an absolute value of log2(fold change) > 1 were designated here as differentially expressed genes (DEGs).

### 2.6. Functional Annotation and Enrichment Analysis

Given the currently incomplete annotation information of cultivated strawberry genome, we annotated the genome-guide assembly transcripts with eggnog-Mapper software against the eggnog database [42]. The GO (Gene Ontology) and KEGG (Kyoto Encyclopedia of Genes and Genomes) annotations of transcripts obtained by eggnog-Mapper were conducted into ClusterProfiler (v3.18.1) for the GO and KEGG enrichment analyses of DEGs' transcripts [43].

The ORF (Open Reading Frame) prediction and translation of transcripts were both performed using the OrfPredictor program (v2.3) [44]. The ensuing protein sequences were then searched against the Pfam database by using the PfamScan program for the annotation of conserved domains [45, 46]. An R script extracted the PfamScan results for a protein conserver domain enrichment analysis of DEGs that was then evaluated statistically using Fisher's exact test.

### 2.7. Survey of FaBBX Transcription Factor Family of Strawberry

Based on our RNA-Seq assembly data and annotation of Pfam database, we conducted a survey of the FaBBX family. Briefly, the proteins containing zf-B_box protein domain (PF00643) were regarded as members of the FaBBX protein family. The domains of FaBBX proteins were annotated by the Pfam database (http://pfam.xfam.org/) [46]. The proteins' domain distribution diagram was visualized using TBtools [47].

## 3. Results

### 3.1. Blue Light Promotes Flowering Induction in Cultivated Strawberry

**Figure 1:**
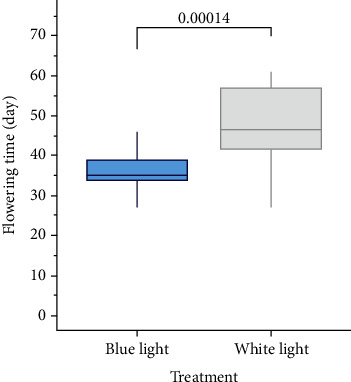
Boxplots of flowering time of strawberry seedlings under blue light treatment and white light treatment.

**Figure 2:**
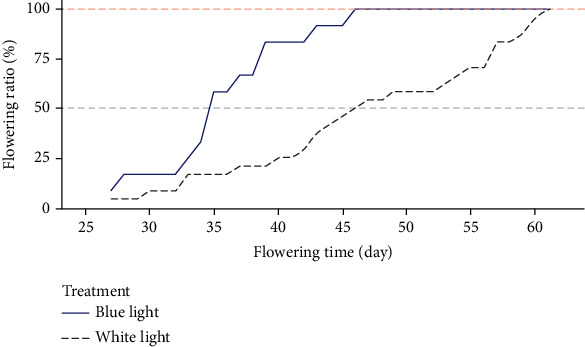
Flowering time of strawberry seedlings under the blue light treatment and white light treatment. Shown is a line plot of the recorded percentage of blooming seedlings.

We observed the flowering time of strawberry seedlings under two light quality treatments. As Figure 1 shows, the blue light treatment significantly advanced strawberry's flowering time. All the strawberry seedlings under blue light treatment bloomed on or before the 46th day after treatment (DAT), whereas at this time only 50% of the seedlings under the white light treatment had bloomed (Figure 2). We sampled the leaves of strawberry seedlings at that time (DAT = 46).

### 3.2. Transcriptome Assembly and Identification of DEGs

Transcriptional regulation is a major way of flowering regulation. To obtain a global understanding of the molecular mechanism underlying the regulation of flowering time as affected by light quality, six cDNA libraries of leaf samples on the 46th DAT under two light quality treatments were generated for RNA sequencing on the Illumina platform. Statistics of the sequencing data are presented in Table 1. A set of raw data (42.41 Gbp) was first generated, from which clean data (37.33 Gbp) with high-quality reads were obtained after data filtering. More than 92.95% of these clean reads had a quality score at the Q30 level (error rate < 0.001); this indicated that the data were robust for subsequent analyses.

In the next analysis, more than 90.18% of clean reads were successfully mapped back to the reference genome data of cultivated strawberry, the latter released recently [37]. As seen in Table 1, the high mapping rate of all sequencing data indicated the reliability of our sequencing data. In addition, this result also demonstrated the suitability of cultivated strawberry genome data for our follow-up transcriptome analysis. All the clean reads were run through the HIASAT2-Stringtie pipeline for a genome-guided transcriptome assembly. In this way, a total of 152 031 transcripts were obtained.

**Figure 3:**
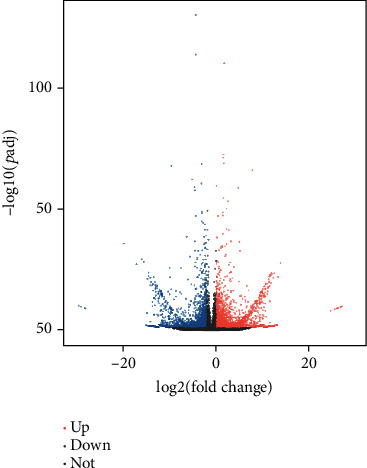
A volcano plot of differentially expressed genes (DEGs) that responded to the blue light treatment (BL). On the *y*-axis is the –log10(*P*adj) value, with the log2 (fold change) plotted on the *x*-axis. The red points are transcripts of significant upregulation (∣fold change∣>1 and *P*adj < 0.05) under BL; the blue points are transcripts of significant downregulation under BL. The gray points denote transcripts of nonsignificant change.

**Figure 4:**
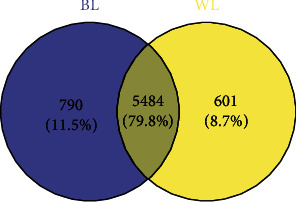
Uniquely expressed genes responsive to the blue light treatment (BL). Shown is a Venn plot of genes expressed only in BL or WL (white light treatment) and those common to both. In the blue area is the number of such genes under BL; in the yellow area is the number of such genes under WL; the gray area is the number of DEGs expressed both in BL and WL.

**Figure 5:**
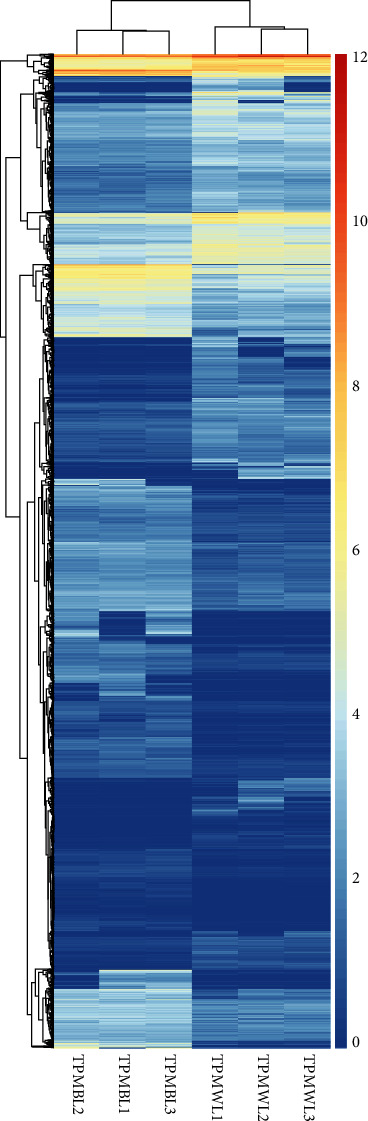
A heatmap of the expression profiles of DEGs. The expression level was normalized using the TPM method, and the color scale bar represents the log10 (TPM + 1). The TPMBL1, TPMBL2, and TPMBL3 correspond to three biological replications under the blue light treatment. The TPMWL1, TPMWL2, and TPMWL3 are the three biological replications under the white light treatment.

The gene expression levels were calculated and normalized using TPM. Pearson's correlation coefficient was calculated between the different samples. A PCA (principal components analysis) of different sample data was used to ensure the reliability of biological replications' data (Figures S1 and S2). To identify the genes responding to the light quality treatments, an analysis of DEGs was performed (using the DESeq2 package in R). As a result, a total of 6765 genes, consisting of 3737 (54.36%) upregulated genes and 3138 (45.64%) downregulated genes, were identified as DEGs between the white vs. blue light treatment (Figure 3, Figure S3). In addition, TMP > 0 was applied as a criterion to define the uniquely expressed genes among the DEGs. This revealed that 601 (8.7%) DEGs were expressed under the white light treatment with a TPM = 0 under the blue light treatment. Conversely, 790 (11.5%) DEGs were identified as uniquely expressed genes under the blue light treatment, these having a TPM = 0 under the white light treatment (Figure 4). The DEGs were hierarchically clustered according to the expression pattern under the two treatments. These results showed a similar gene expression pattern for the same treatment biological replications, in that the DEGs from BL and WL clustered together, respectively (Figure 5).

### 3.3. Gene Function Enrichment Analysis for DEGs

To better understand the gene functions of the DEGs, we conducted a systematic functional annotation of all assembled transcripts of these DEGs and their enrichment analysis (Table S1). The GO enrichment analysis showed that the DEGs were significantly enriched in three main GO categories of “cellular component,” “molecular function,” and “biological process.” In this respect, the “biological process” category, with 73 GO terms, was the largest GO category, followed by the “molecular function” category, with 18 GO terms, leaving the “cellular component” category the least represented, containing only 5 GO terms (Figure S4, Table S2).

**Figure 6:**
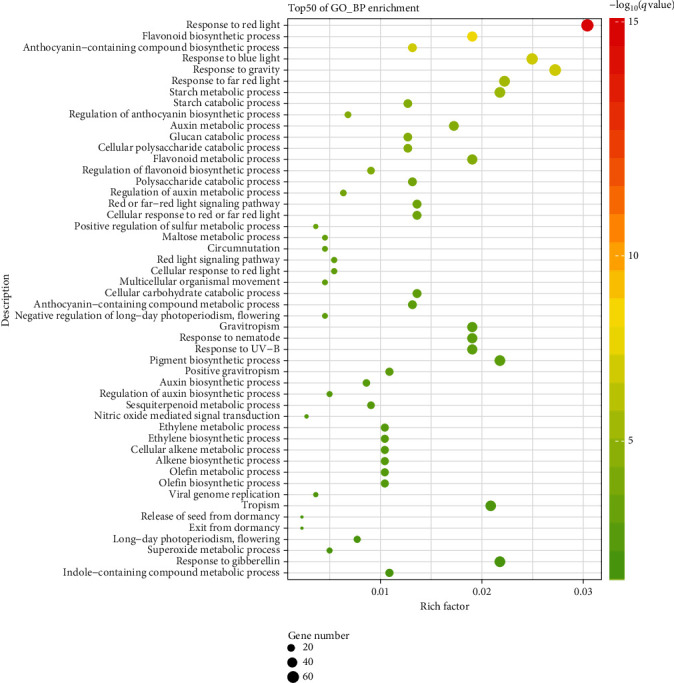
GO enrichment analysis of DEGs. Shown are the top 50 enriched GO terms of the “biological process” category. On the *y*-axis is the description of the corresponding GO terms.

In the “biological process” category (Figure 6, Table S2), “response to red light” (GO: 0010114), “flavonoid biosynthetic process” (GO: 0009813), and “anthocyanin-containing compound biosynthetic process” (GO: 0009718) were the top three enriched GO terms according to the –log10(*q* value). As expected, the GO terms involved in the light signal response were enriched in our study's DEGs, such as “response to blue light” (GO: 0010114), “response to far-red light” (GO: 0010218), “red or far-red light signaling pathway” (GO: 0010017), “red light signaling pathway” (GO: 0010161), and “response to UVB” (GO: 0010224). Besides, several GO terms related to secondary metabolism also were enriched, such as “regulation of anthocyanin biosynthetic process” (GO: 0031540) and “flavonoid metabolic process” (GO: 0009812). Two GO terms, “negative regulation of long-day photoperiodism, flowering” (GO: 0048579) and “long-day photoperiodism, flowering” (GO: 00485 74), both involved in the flowering process, were also enriched in our analysis (Figure 6, Table S2).

**Figure 7:**
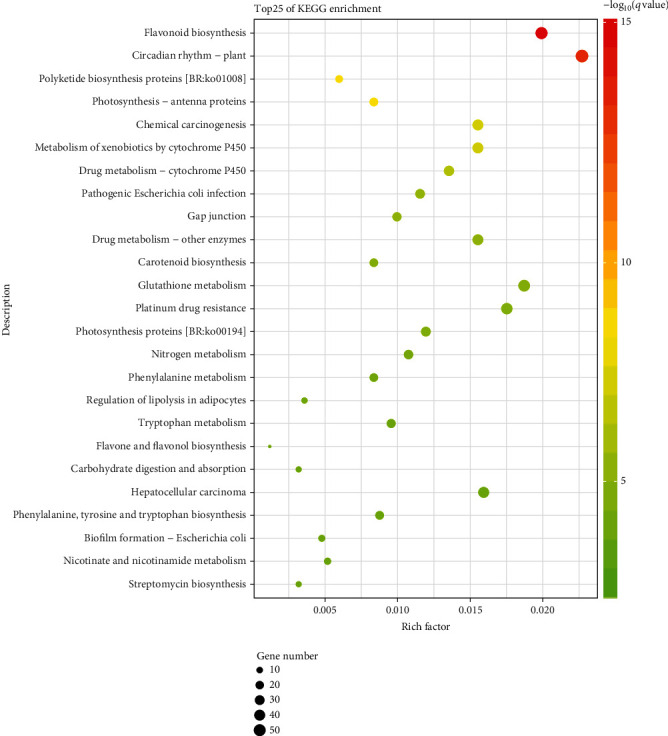
KEGG enrichment analysis of DEGs. Shown are the top 25 KEGG pathways enriched in DEGs. On the *y*-axis is the description of the corresponding KEGG pathways.

The KEGG enrichment analysis provided more information on DEGs at the metabolic pathway level. Specifically, DEGs were significantly enriched in 71 pathways (Table S3). As Figure 7 shows, the “flavonoid biosynthesis” (ko00941) was the most significantly enriched pathway, containing 50 transcripts in total. The “circadian rhythm” (ko04712) pathway was also significantly enriched, comprising 57 transcripts.

**Figure 8:**
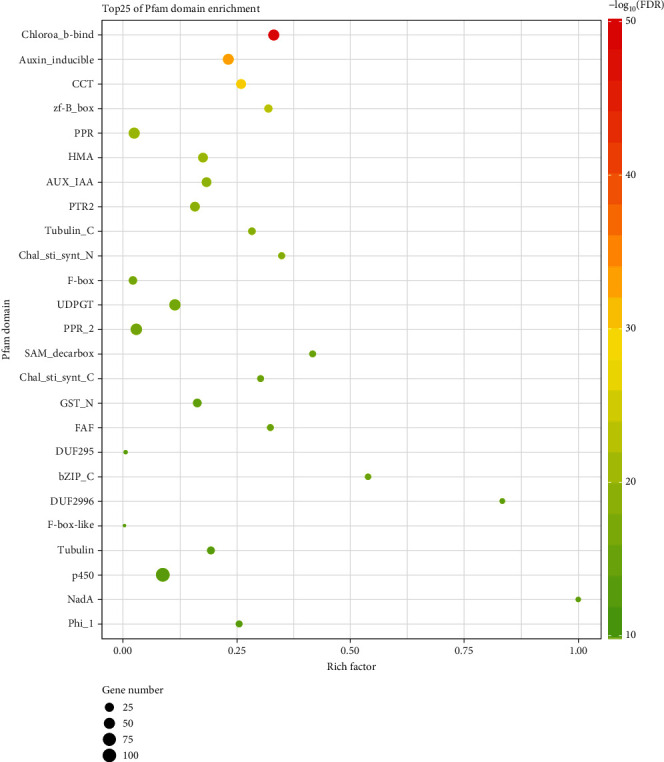
A conserved domain enrichment analysis of DEGs. Shown are the top 25 protein conserved domain enrichments found, based on their annotation using the Pfam database. On the *y*-axis are the names of the corresponding conserved domains. The rich factor is the ratio of the number of DEGs to the total number of genes in the annotation cluster. The gene number is the number of DEGs in the annotation cluster. The color scale bar indicates the –log10 (*q*-value); the *q* value is the *P* value adjusted by the “BH” method.

The identification of domains of proteins can provide insight into the respective functioning of proteins. The Pfam database is a large collection of protein domains and protein families. Given that a particular protein domain structure gives rise to a particular protein function, an enrichment analysis based on Pfam database annotation was conducted here. As Figure 8 shows, those proteins containing the Chloroa_b-bind domain (PF00504) were the most significantly enriched. In addition, transcription factors play important roles in signal transduction by operating as gene expression regulators. In this study, the proteins containing the zf-B_box domain were significantly enriched for 23 DEGs (Figure 8). Additionally, the zf-B_box protein domain (PF00643) is the characteristic functional domain of the BBX transcription factor family in plants.

### 3.4. Altered Gene Expression Involved in Light Signal Response

Light as a critical environmental signal affects many plant physiological processes. The various light-signal transduction pathways have been identified in the model plant *Arabidopsis*. As our above results for GO enrichment showed, the DEGs were significantly enriched in eight GO terms (“GO:0010114,” “GO:0009637,” “GO:0010218,” “GO:0010017,” “GO:0071489,” “GO:0010161,” “GO:0071491,” and “GO:0071482”) known to be related to the perception and transduction of light signals. To further investigate gene expression patterns and functions of DEGs involved in the light response, we examined the expression data of 147 DEGs subjected to the above eight GO terms and their functional annotation based on the Uniref90 database (Table S4). Among these transcripts, 72 (48.97%) transcripts were found upregulated under the blue light treatment while 75 (51.02%) transcripts were downregulated (Figure S5, Table S4).

**Figure 9:**
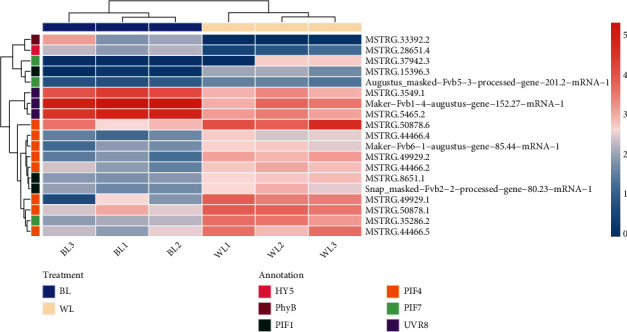
Expression heatmap of those genes involved in light perception and transduction. The color scale bar indicates the log10 (TPM + 1). The transcripts are annotated using different colors. The BL1, BL2, and BL3 are the three biological replications under the blue light treatment. The WL1, WL2, and WL3 are the three biological replications under the white light treatment.

Light receptor proteins trigger downstream light signal transduction in plants by perceiving the external light environment. Plants can sense distinct external light quality through several classes of light receptors, such as cryptochromes (CRYs), phytochromes (Phys), phototropins (PHOTs), and ultraviolet-B receptors (UVR8s). Among these light receptors, two types were affected by the blue light treatment in our study (Figure 9). The transcript encoding a Phy protein (MSTRG.33392.2) and three transcripts encoding UVR8 (maker-Fvb1-4-augustus-gene-152.27-mRNA-1, MSTRG.5465.2, and MSTRG.3549.1) were significantly promoted by blue light treatment. By contrast, the expression of transcripts encoding light receptors responsible for other types of light signal pathways in plants showed nonsignificant changes between the two treatments.

The ELONGATED HYPOCOTYL5 (HY5), a bZIP transcription factor protein, was reported to be a crucial hub in the light signal transduction network of plants. In our study, the expression level of a transcript (MSTRG.28651.4) encoding a homologue of HY5 was promoted by the blue light treatment (Figure 9). Furthermore, phytochrome-interacting factors also responded to blue light in this study. We found four transcripts (MSTRG.8651.1, MSTRG.15396.3, MSTRG.8651.1, and snap_masked-Fvb2-2-processed-gene-80.23-mRNA-1) encoding PIF1 proteins, eight transcripts (MSTRG.50878.1, MSTRG.44466.2, MSTRG.44466.4, MSTRG.44466.5, MSTRG.49929.1, MSTRG.49929.2, MSTRG.50878.6, and maker-Fvb6-1-augustus-gene-85.44-mRNA-1) encoding PIF4 proteins, and three transcripts (MSTRG.35286.2, augustus_masked-Fvb5-3-processed-gene-201.2-mRNA-1, and MSTRG.37942.3) encoding PIF7 proteins. Expression levels of transcripts encoding PIFs were repressed by the blue light treatment (Figure 9).

### 3.5. Genes Involved in the Circadian Rhythm Floral Induction

In this study, blue light exposure evidently affected the flowering time of the experimental strawberry seedlings. Our enrichment analysis results based on GO and KEGG annotations indicated that the blue light treatment modified 66 transcripts whose expression level could be classified into two GO terms (“GO:0048579” and “GO:0048574”) and 1 KEGG pathway (ko04712). Overall, 20 transcripts (30.30%) were identified as downregulated by blue light while 46 transcripts (69.69%) were upregulated (Figure S6, Table S5).

**Figure 10:**
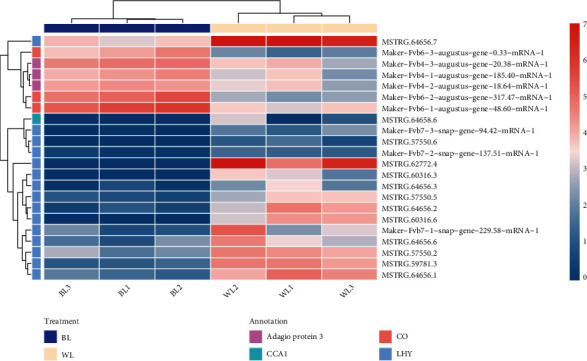
Expression heatmap of those genes involved in the circadian rhythm pathway. The color scale bar indicates the log10 (TPM + 1). The transcripts are annotated using different colors. The BL1, BL2, and BL3 are the three biological replications under the blue light treatment. The WL1, WL2, and WL3 are the three biological replications under the white light treatment.

The FvCO (CONSTANT) protein plays an important role in the floral induction of wild strawberry [33]. In this study, three transcripts (maker-Fvb6-2-augustus-gene-317.47-mRNA-1, maker-Fvb6-3-augustus-gene-0.33-mRNA-1, and maker-Fvb6-1-augustus-gene-48.60-mRNA-1) that encode CO protein homologues in strawberry were identified from DEGs which showed upregulated expression pattern under the blue light treatment. Similarly, we found the expression levels of three other transcripts (maker-Fvb4-3-augustus-gene−20.38-mRNA-1, maker-Fvb4-1-augustus-gene-185.40-mRNA-1, and maker-Fvb4-2-augustus-gene-18.64-mRNA-1) encoding Adagio protein 3, a homologue of FKF1, also promoted by the blue light treatment. Our RNA-Seq results also detected the downregulation of 16 transcripts involved in circadian rhythm floral induction pathways. Of these transcripts, the transcript MSTRG.64658.6 encodes a homologue of CCA1 (Circadian Clock Associated 1) protein and the remaining 15 transcripts were annotated as coding LHY (LATE ELONGATED HYPOCOTYL) proteins (Figure 10).

### 3.6. Survey of BBX Transcription Factors

**Figure 11:**
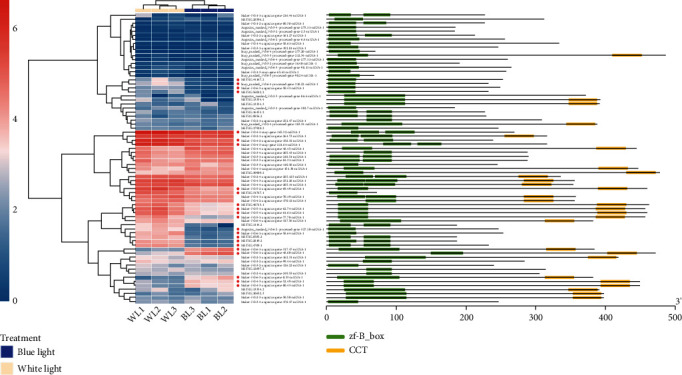
Survey of the BBX transcription factor family. The heatmap on the left conveys the expression levels of the BBXs. The color scale bar in the lower left corner indicates the log10 (TPM + 1). The red dots before the transcript names denote the case of significant differential expression, based on the present RNA-Seq analysis. The schematic on the right summarizes the domain distribution of BBX proteins encoded by the transcripts. The green and yellow blocks correspond to the B-box domain and CCT domain, respectively.

Many transcription factors have been demonstrated to participate in light signal transduction and flowering regulation. Our above enrichment analysis using the Pfam database annotations suggested that the BBX protein family could figure prominently in floral induction under blue light. To investigate this further, we conducted a comprehensive survey of BBX transcription factors based on both the Pfam annotations and RNA-Seq assembly data. These results uncovered a total of 72 transcripts encoding BBX proteins, each containing at least one zinc finger B-box conserved domain (Figure 11).

There were 23 significant differentially expressed transcripts found encoding a BBX protein, of which 18 were downregulated and 5 were upregulated under blue light. The annotation results according to the TAIR database (https://www.arabidopsis.org/) indicated that the 23 proteins were homologues of AtCO, AtBBX15, AtBBX19, AtBBX24, and AtBBX29 (Table S6). In terms of the expression levels of these transcripts, maker-Fvb4-4-snap-gene-165.32-mRNA-1 encoding a homologue of AtBBX24 exhibited the highest expression level. The transcript MSTRG.2819.2, which encodes a homologue protein of AtBBX19, showed differential expression that was the most significant statistically, with a *P*adj value of 2.33*E* − 114. In terms of magnitude, the transcript snap_masked-Fvb6-4-processed-gene-318.22-mRNA-1 encoding a homologue protein of AtBBX29 underwent the most significant change in expression level, with a log2 (fold change) value of –4.41.

## 4. Discussion

### 4.1. Effect of Blue Light on Strawberry

Light is a crucial environmental factor affecting multiple aspects of plant growth. With the widespread usage of plastic greenhouse and growth chambers in protected cultivation, LED (light-emitting diodes) are now widely used to provide the primary light source or as supplementary illumination, in addition to ambient light, as a tool for fine-tuning the light conditions of the plant growth environment [48, 49]. In previous work from our laboratory, applying a blue light treatment in the growth chamber was able to significantly increase the total anthocyanin content and change the anthocyanin profile of strawberry fruits [50]. LED light resources of light quality are often used in breeding systems because optimizing light quality could improve breeding timelines by accelerating plant growth. The ratio of red to blue light is a vital factor for flowering [51]. Blue light was shown to promote woodland strawberry's flowering when applied in an LED system [5]. In this study, cultivated strawberry seedlings were exposed to blue light or white light, also using LED as the light resource. The results showed that the blue light treatment significantly promotes flowering in cultivated strawberry when compared with the white light treatment. This finding is similar to research reported on woodland strawberry and petunia [52].

Besides promoting plant floral initiation, light quality can affect other plant physiological processes. For example, chlorophyll content of grape leaves was significantly higher in plantlets grown under blue light than white light [53]. In our study, enrichment analysis based on the Pfam database annotation for the conserved protein domain also demonstrated that gene expression levels of transcripts encoding proteins containing chloroa_b binding domain were affected by blue light.

Work demonstrated that blue light enhances the production of secondary metabolites, such as phenolics and flavonoids, in callus cultures of *Stevia rebaudiana*. Our laboratory's previous research also found effects on the secondary metabolism of strawberry fruit under different light quality treatments [54]. In the present study, the DEGs were significantly enriched in GO terms and KEGG pathways, indicating that the blue light treatment may also have a similar effect on the leaves of seedlings. The light was reported to affect the levels of phytohormone in plants. The indoleacetic acid (IAA) content of Norway spruce (*Picea abies* (L.) Karst.) tree seedlings under blue light LED illumination was significantly higher than those illuminated by red light; their transcriptome findings showed that blue light modified the gene expression involved in auxin-response transduction [55]. Likewise, from an annotation and enrichment analysis of DEGs, we found some related to auxin metabolism that were significantly enriched in strawberry. Hence, our study obtained a similar result in that strawberry seedlings are capable of responding to blue light via the mediation of auxin metabolism. However, whether this changed auxin metabolism is related to the flowering of strawberry awaits further investigation in future studies.

### 4.2. The Light Transduction Network Involved in response to Blue Light

As mentioned above, light is an essential factor regulating various processes in plants. From research using the model plant *Arabidopsis*, a comprehensive signaling network associated with light signal sensing and transduction has been established.

Plants sense light signals via various photoreceptor proteins. The FKF1 protein functions as a blue light receptor harboring a LOV domain, which can conduct the light signal to the flowering pathway by regulating the stability of the CDF1 protein [56, 57]. In our experiment, the gene expression of FKF1 in strawberry was influenced by the blue light treatment, as a significant increase in the expression level of FKF1 occurred in the blue light treatment. The UVR8 protein is the photoreceptor of UVB radiation; in the absence of UVB radiation, UVR8 protein is a dimer formed by an electrostatic interaction of monomer proteins. The dissociation of UVR8 dimer, caused by UVB radiation, enables the monomeric UVR8 to initiate signal transduction of UVB by interacting with the COP1 protein [58]. It has been proposed that UVR8 mediates UVB light signal inputs to the central oscillator and modulates gene expression related to the circadian rhythm in plants [59]. In previous work [60], blue light altered the expression level of four transcripts encoding UVR8 proteins. Among them, three transcripts were downregulated by blue light, and only one transcript showed an opposite expression pattern. By contrast, all the transcripts encoding UVR8 were upregulated under the blue light treatment in our results for strawberry seedlings; this difference may be due to different sampled tissue types between the two studies. The phytochrome proteins are responsible for plants' perception of red or far-red light. In *Arabidopsis*, a total of five phytochromes have been identified, known as PhyA–PhyE [61]. Research using *Arabidopsis* has demonstrated that PhyB protein is involved in shade avoidance responses invoked under blue light illumination [62, 63], with more recent research showing that PhyB can play a pivotal role in response to blue light [64]. From the current study's research, a transcript encoding a homologue protein corresponding to PhyB protein in *Arabidopsis* was identified as a DEG. This result suggests that the complex signal network of strawberry response to blue light might involve multiple light signal pathways.

The bZIP-type transcription factor HY5 is an important hub of the light signal network. The HY5 protein acts downstream of multiple families of the photoreceptors and regulates target genes by binding to their promoters. One analysis identified more than 3000 chromosomal sites qualifying as putative AtHY5 binding targets [65]. Meanwhile, the transcription level of the *AtHY5* gene can be differentially regulated by variation in the light quality signals as well. In several different species, the expression level of *HY5* has been found regulated by different light qualities [60, 66, 67]. In our study, the expression level of *HY5* was promoted by blue light, which agrees with research findings for Longan (*Dimocarpus longan* Lour.) embryonic calli [68]. Furthermore, a relationship between HY5 protein and circadian rhythm genes was reported when using the *hy5* mutant as plant material [69, 70]. More recently, the circadian clock-related function of HY5 and its homologue HYH (HY5 Homologue) was clarified: HY5/HYH mediates blue light signaling into the circadian oscillator system in plants via transcriptional regulation of the clock genes [71]. Hence, we speculate that the altered expression of HY5 might contribute to shifting the flowering time of strawberry seedlings earlier under the blue light treatment.

The so-called PIFs (phytochrome-interacting factors) are a class of basic helix-loop-helix domain-containing transcription factors that interact physically with phytochromes [61]. Rapid progress in understanding the gene functioning of PIFs has been made in the last decade, which has revealed that the family of PIF proteins plays a central role in light signaling transduction and also participates in various processes of plant physiology. Hitherto, a total of eight PIF proteins with the APB motif were identified [72]. In our study, 15 transcripts encoding three homologues of PIFs were identified as DEGs, indicating that PIF proteins from strawberry contribute to its blue light response as well. The PIF1 protein can be rapidly phosphorylated, ubiquitylated, and degraded under blue light conditions [73]. In our research, the expression level of *PIF1* displayed a downregulated pattern. However, the PIF1 was deemed a regulator of flowering in *Arabidopsis* [74]. Given that the previous work demonstrated PIF1 functions as a negative regulator of chlorophyll biosynthesis to optimize deetiolation of *Arabidopsis* seedlings [75, 76], we speculate that the PIF1 protein of strawberry may also act as a regulator, but one involved in governing the chlorophyll biosynthesis in strawberry seedlings' response to blue light. The PIF4 protein can operate as a partner protein of CRY proteins mediating light signaling under blue light, in that the interaction of CRYs and PIF4 modulates transcriptional activity of PIF4 [64, 77]. We found the *PIF4* was downregulated under the blue light treatment in our study. AtPIF4 is a positive regulator promoting flowering in the thermosensory flowering response according to previous *Arabidopsis* research [78]. However, the lowered expression level of *PIF4* under blue light condition compared to its gene expression under white light suggests that *PIF4* may not partake in the modulation of flowering time in strawberry plants exposed to blue light. We also found that *PIF7* was regulated by blue light treatment as a downregulated gene. Nevertheless, the biological function of AtPIF7 remains unclear.

Based on our analyses, we propose that blue light may affect flowering time via photoreceptors and signal transduction components whose altered activity leads to changes in downstream gene expression.

### 4.3. The Roles of Genes Related to Circadian Rhythm Floral Induction

The circadian clock provides essential timing information to ensure plants' optimal growth to external environmental conditions, by processing different light wavelengths, intensities, and photoperiodic duration for the internal clock-setting mechanism [79]. As our KEGG enrichment analysis showed, some of the DEGs responsive to the blue light treatment were significantly enriched in the plant circadian rhythm pathway.

In the past two decades, much research has established a regulation network of the circadian rhythm in the model plant *Arabidopsis*. Three *Arabidopsis* genes that may encode core components of the circadian central oscillator are CIRCADIAN CLOCK ASSOCIATED 1 (CCA1), LATE ELONGATED HYPOCOTYL (LHY), and TIMING OF CAB 1 (TOC1) [80]. As key components of the circadian rhythm feedback loop, the AtLHY1 and the AtCCA1 have been identified as MYB transcription factors [70]. In later research, Nagel et al. identified over 1000 directly transcriptional regulatory genes related to myriad biological processes and stress responses, using ChIP followed with deep sequencing [81]. AtCCA1 is believed to function as a close linkage between the flowering time, the circadian rhythm, and floral induction pathway, by modulating the gene expression of the AtCO-AtFT pathway [82]. Constitutive expression of the AtCCA1 protein in transgenic plants abolished the circadian rhythm of several genes with dramatically different phases, and these plants featured a phenotype characterized by longer hypocotyls and delayed flowering [13]. Work by Lu et al. showed that AtCCA1 binds directly to *AtGI* promoter to repress its expression [83]. The LHY protein is closely related to CCA1 protein, and the functions of AtCCA and AtLHY are partially redundant [80]. The AtLHY mutant (*lhy*) displayed a phenotype of late flowering under a long-day condition [13]. In addition, overexpression or silencing of *NaLHY* from *Nicotiana attenuata* altered this plant's timing of flower opening [84]. Given that our results showed the downregulated expression pattern of both *CCA1* and *LHY* under the blue light treatment, their altered gene expression levels may have affected the flowering time of strawberry in our research by converting the light signal into a floral induction pathway.

### 4.4. Roles of the BBX Family in Regulating Flowering Time

In the light transduction and floral induction pathway, transcription factors play critical roles by regulating gene expression to adequately respond to external environmental cues. The transcription factors in plants can be classified into different families based on their DNA-binding domains. To date, over 300 000 transcription factors from 165 plant species are classified into 64 families according to their annotation in the Pfam and PlantTFDB databases [85, 86]. Here, we carried out the conserved domain annotation of DEGs based on the Pfam database and an enrichment analysis. Our result showed that the transcription factors having a zf_B-box conserved domain were significantly enriched among the identified DEGs.

The BBX family represents a subgroup of zinc finger proteins that contain at least one B-box domain. In *Arabidopsis*, 32 BBX proteins are known and categorized into five structural groups depending on the presence of B-box domains and CCT domains [87, 88]. More recently, researchers have identified 25 BBX genes in pear (*Pyrus bretschneideri* Rehd.) and 64 BBX genes in apple (*Malus domestica* Borkh.) [89, 90]. In our previous research, 21 BBX genes were identified from woodland strawberry (*F. vesca*) [91]. In the present study, we identified a total of 72 transcripts encoding BBX proteins in cultivated strawberry based on its genome data and RNA-Seq data from the experiment.

The BBX proteins mainly participate in several plant physiology processes including photomorphogenesis, flowering regulation, shade avoidance responses, stress responses, and hormonal signaling networks [87]. Here, we identified 23 transcripts encoding BBX proteins among the DEGs responding to the blue light treatment. These 23 proteins were homologues of five AtBBXs. The AtCO/AtBBX1 from *Arabidopsis*, which contains two tandem B-box domains and a CCT domain in N-terminal of the protein, was the first BBX protein identified in plants. When compared with the wild type, the AtCO mutant plants (*co*) flower later whereas the overexpression lines of AtCO show an early flowering phenotype [92, 93]. The AtCO protein promotes *AtFT* expression by binding to the promoter region of the *AtFT* gene via the CCT motif, and the expression of AtCO is altered by day length and light quality in *Arabidopsis* [9, 94, 95]. Blue light can also promote *AtCO*'s functioning at the transcription level and posttranscript level [96]. Furthermore, Kurokura et al. showed that FvCO, a homologue of AtCO, regulated the flowering time in wild strawberry; silencing lines of *FvCO* flowered late in the long-day-flowering accession “Hawaii-4,” while the lines overexpressing *FvCO* flowered earlier [33]. In our study, the gene expression levels of homologue genes of *AtCO* were promoted by the blue light treatment, suggesting that the CO genes in cultivated strawberry mediate its early flowering when exposed to blue light.

In this study, two transcripts encoding homologue proteins of AtBBX15/AtCOL16 were identified as upregulated DEGs. These proteins contain one B-box domain and a CCT domain; both are classified members of group III of the BBX protein family. Recently reported research on the function of a gene encoding a homologue of AtCOL16 found PhCOL16 involved in chlorophyll accumulation [97]. Meanwhile, our GO and KEGG analyses demonstrated that the blue light might have influenced chlorophyll biosynthesis in strawberry. Taken together, we speculate that these transcripts encoding homologues of AtBBX15 are regulated by blue light and play a part in chlorophyll accumulation under blue light illumination.

The AtBBX19 protein harbors two B-box domains at the N terminus but lacks the CCT domain. The AtBBX19 protein can physically interact with the AtCO protein and repress the function of AtCO. In transgenic *Arabidopsis*, *AtBBX19* overexpression lines reduced the *AtFT* mRNA levels while the T-DNA insertion lines (bbx19-1 and bbx19-2) showed a late flowering phenotype [98]. And in our results, blue light promoted the gene expression of *FaBBX19*, which suggests that the FaBBX19 protein in strawberry may function similarly to AtBBX19. Evidently, the functional details of FaBBX19 activity in strawberry under blue light deserve further investigation.

The AtBBX24 protein, a group IV member of the AtBBX family, has a characteristic one B-box domain and a CCT domain. Here, we found four transcripts in strawberry encoding homologue proteins of AtBBX24. In *Arabidopsis*, the encoding gene's expression level was regulated by various external environmental cues, such as UVB illumination, red light, and cold stress [87]. The AtBBX24 protein has been demonstrated to participate in light signal transduction and photomorphogenesis by interacting with AtCOP1 [99]. In this study, the expression levels of four transcripts that encode AtBBX24 homologue proteins from strawberry were promoted under the blue light treatment. In other research, the *AtBBX4* mutant lines (*sto-1*) flowered later than did the wild-type lines under short-day growing conditions, while overexpression of *AtBBX4* in *Arabidopsis* induced an early flowering phenotype under both long-day and short-day conditions [100]. In a different plant, the *CmBBX24* from *Chrysanthemum morifolium* was found to operate as an association hub between its flowering time and stress tolerance; transgenic lines of *C. morifolium* with suppressed expression of *CmBBX24* flowered earlier than did wild-type counterparts [101]. The altered gene expression patterns of BBX24 under the blue light treatment may have affected the flowering of strawberry in the present study.

In our research, six transcripts encoding homologue proteins of AtBBX29 were identified as significant downregulated DEGs under the blue light treatment. The AtBBX29 was classified as a member of group V of the AtBBX family, which contains only one B-box domain. The group V of the BBX family comprises seven members (AtBBX26–AtBBX32) [88]. In comparison with the other BBX family groups, there is less research done focusing on the function of members of group V of the BBX family. The AtBBX32 was demonstrated to act antagonistically to HY5, and it negatively mediated gene expression repression to maintain dark adaptation [102]. Later, the AtBBX32 was found regulated by the circadian clock pathway, and the interaction between AtBBX4 and AtBBX32 negatively regulated flowering via the repression of *AtFT* [103]. Further, the AtBBX30 and AtBBX31 also were shown to be involved in the regulation of photomorphogenesis in *Arabidopsis* plants [104]. However, the function of AtBBX29 remains unknown. In this study, the expression level of *FaBBX29* that encodes a homologue of AtBBX29 in strawberry was remarkably repressed under the blue light treatment. Accordingly, we speculate that the *FaBBX29* may be a negative regulator of flowering responding to different light quality treatments. However, the functional details of *FaBBX29* in the mechanisms of flowering time regulation need further investigation, ideally by employing genetic and biochemistry techniques in tandem.

## 5. Conclusions

In the present study, the blue light treatment promoted the flowering of cultivated strawberry seedlings. We further analyzed the global transcriptome of their leaves under two different light quality treatments and provided an overview of the flowering regulation of blue light at the gene expression level. The identification and annotation of DEGs suggested that blue light quality could stimulate the light signal transduction pathway. The altered gene expression of BBX transcription factors participates in the regulation of flowering time. Notably, the *FaBBX29* gene, belonging to the FaBBX family, may figure prominently in the process that regulates flowering time for this valuable crop.

## Figures and Tables

**Table 1:** Summary statistics of the sequencing data.

Data name	Raw data (Gbp)	Clean data (Gbp)	Clean sequence	Q30 (%)	Mapping rate (%)
WL1_R1	3.49	3.07	23286017	94.23	92.50
WL1_R2	3.49	3.07	23286017	93.21	
WL2_R1	3.57	3.14	23803445	94.20	92.40
WL2_R2	3.57	3.14	23803445	92.95	
WL3_R1	3.64	3.20	24249787	94.33	92.17
WL3_R2	3.64	3.20	24249787	93.04	
BL1_R1	3.51	3.09	23416747	94.18	92.05
BL1_R2	3.51	3.09	23416747	93.58	
BL2_R1	3.40	2.99	22645767	94.20	90.18
BL2_R2	3.40	2.99	22645767	93.36	
BL3_R1	3.60	3.17	24011737	94.21	92.19
BL3_R2	3.60	3.17	24011737	93.25	

Notes: Q30: percentage of bases with a Phred value > 30; WL1, WL2, WL3 and BL1, BL2, BL3 represent three biological replication samples from white light treatment and blue light treatment, respectively; the suffixes _R1 and _R2 denote the paired data generated by Illumina paired-end sequencing. Gbp: giga base pair.

## Data Availability

The NCBI SRA data used to support the finding of this study may be accessible with the following link: https://www.ncbi.nlm.nih.gov/bioproject/PRJNA698363.

## Supplementary Materials

Supplementary Materials Figure S1: a PCA plot of samples from different treatments and replications. Figure S2: a Pearson correlation heatmap of samples from different treatments and replications. Figure S3: a pie chart illustrating the percentage of DEGs. Figure S4: a bubble chart visualizing the top 50 enriched GO terms in DEGs. Figure S5: a heatmap of gene expression involved in the light signaling pathway. Figure S6: a heatmap of gene expression which is involved in the circadian rhythm floral induction. Table S1: the expression levels and the function annotations of DEGs. Table S2: the list of top 50 enriched GO terms in DEGs. Table S3: the list of enriched KEGG pathways in DEGs. Table S4: the expression levels of genes involving in light perception and transduction. Table S5: the expression levels of genes involving in the circadian rhythm floral induction pathway. Table S6: the expression levels and annotations of BBX genes in DEGs.
