# Supplementary figures and images for: Transcriptome Profile Analysis of Strawberry Leaves Reveals Flowering Regulation under Blue Light Treatment
Source: Int J Genomics. 2021 Jun 12;2021:5572076. doi: 10.1155/2021/5572076 (PMC8216796; doi:10.1155/2021/5572076)

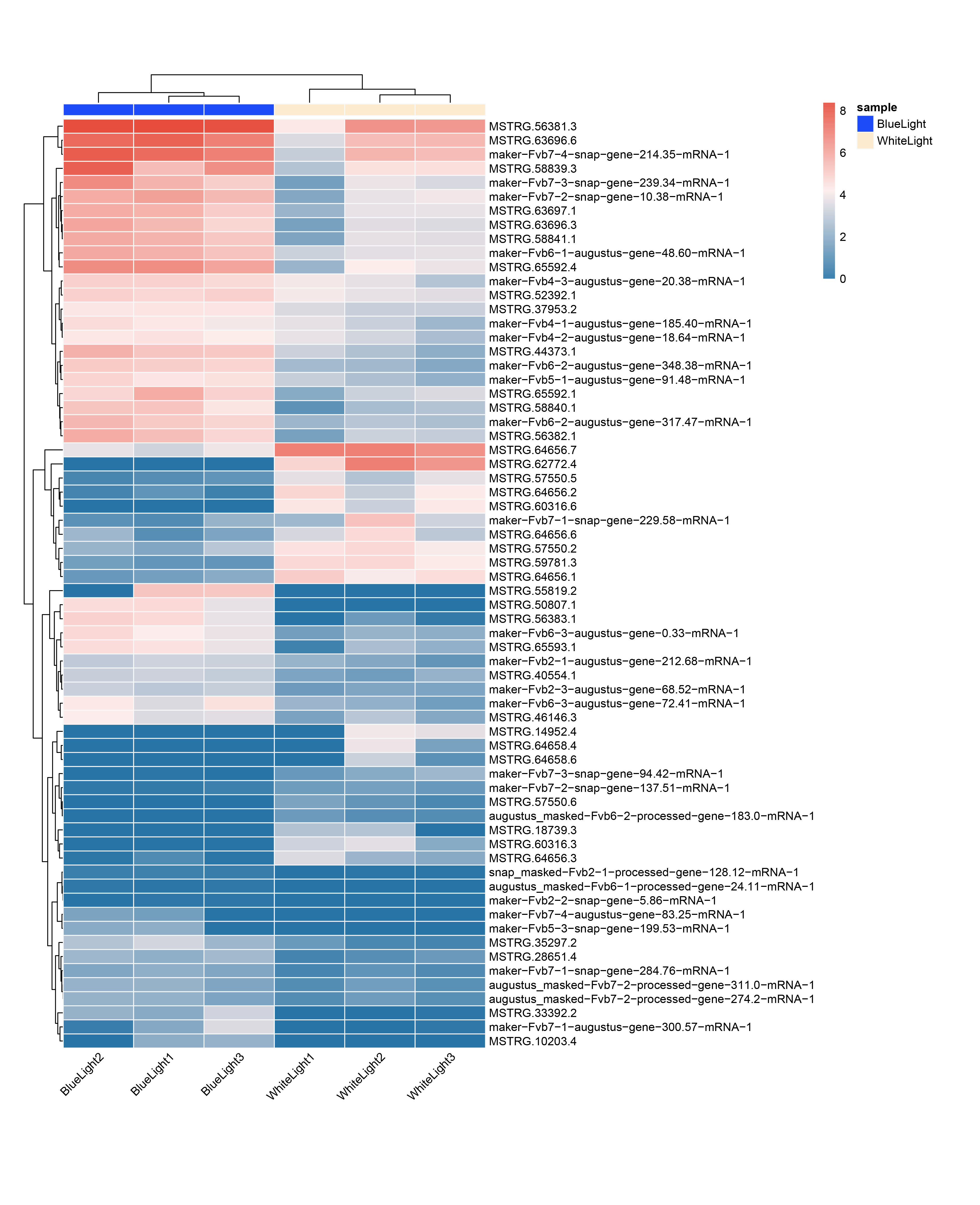

Supplement: Supplementary Materials — Figure S1: a PCA plot of samples from different treatments and replications. Figure S2: a Pearson correlation heatmap of samples from different treatments and replications. Figure S3: a pie chart illustrating the percentage of DEGs. Figure S4: a bubble chart visualizing the top 50 enriched GO terms in DEGs. Figure S5: a heatmap of gene expression involved in the light signaling pathway. Figure S6: a heatmap of gene expression which is involved in the circadian rhythm floral induction. Table S1: the expression levels and the function annotations of DEGs. Table S2: the list of top 50 enriched GO terms in DEGs. Table S3: the list of enriched KEGG pathways in DEGs. Table S4: the expression levels of genes involving in light perception and transduction. Table S5: the expression levels of genes involving in the circadian rhythm floral induction pathway. Table S6: the expression levels and annotations of BBX genes in DEGs. [file 5572076.f1.zip › Supplemental Files/FigureS6.tif]

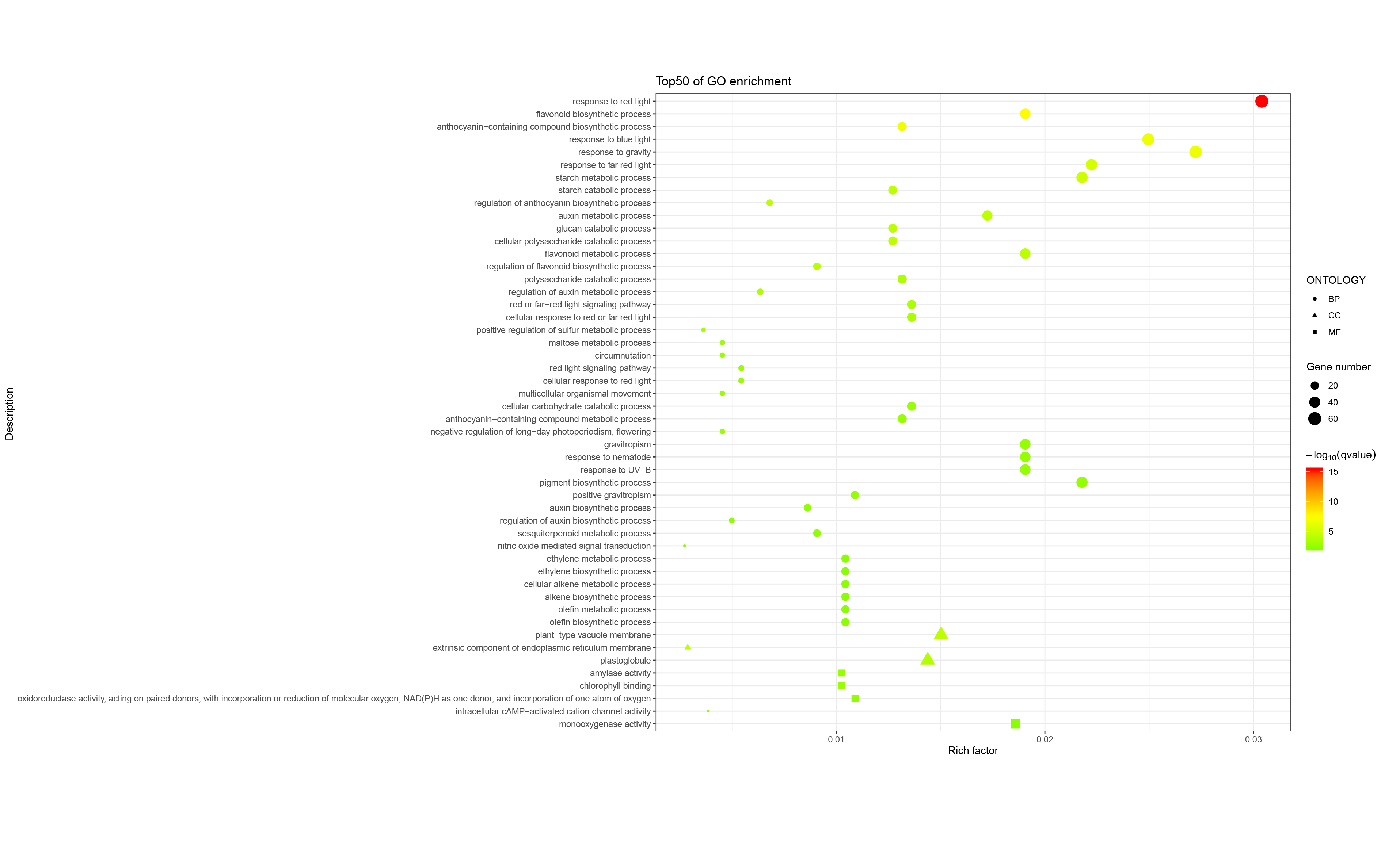

Supplement: Supplementary Materials — Figure S1: a PCA plot of samples from different treatments and replications. Figure S2: a Pearson correlation heatmap of samples from different treatments and replications. Figure S3: a pie chart illustrating the percentage of DEGs. Figure S4: a bubble chart visualizing the top 50 enriched GO terms in DEGs. Figure S5: a heatmap of gene expression involved in the light signaling pathway. Figure S6: a heatmap of gene expression which is involved in the circadian rhythm floral induction. Table S1: the expression levels and the function annotations of DEGs. Table S2: the list of top 50 enriched GO terms in DEGs. Table S3: the list of enriched KEGG pathways in DEGs. Table S4: the expression levels of genes involving in light perception and transduction. Table S5: the expression levels of genes involving in the circadian rhythm floral induction pathway. Table S6: the expression levels and annotations of BBX genes in DEGs. [file 5572076.f1.zip › Supplemental Files/FigureS4.tif]

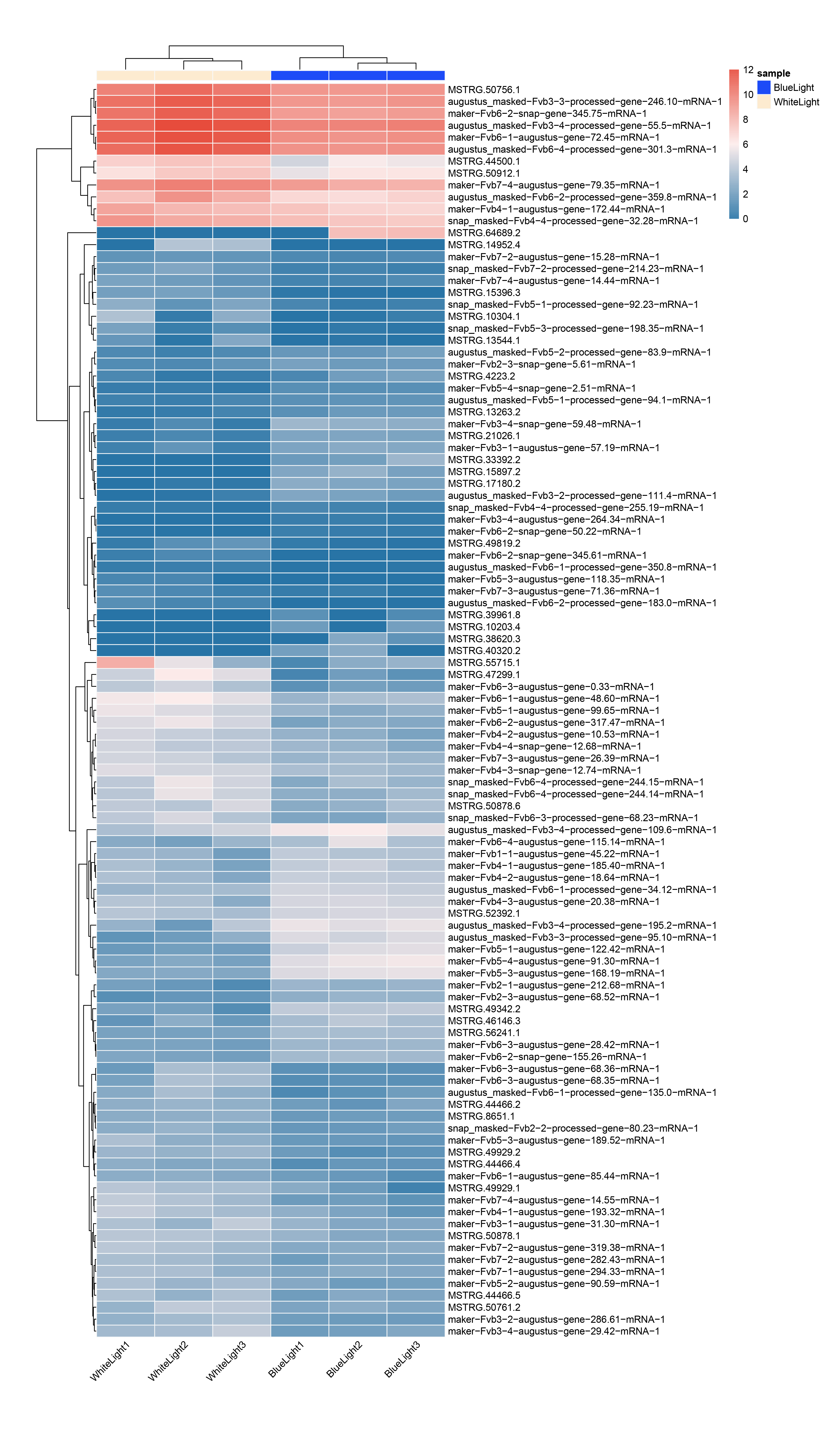

Supplement: Supplementary Materials — Figure S1: a PCA plot of samples from different treatments and replications. Figure S2: a Pearson correlation heatmap of samples from different treatments and replications. Figure S3: a pie chart illustrating the percentage of DEGs. Figure S4: a bubble chart visualizing the top 50 enriched GO terms in DEGs. Figure S5: a heatmap of gene expression involved in the light signaling pathway. Figure S6: a heatmap of gene expression which is involved in the circadian rhythm floral induction. Table S1: the expression levels and the function annotations of DEGs. Table S2: the list of top 50 enriched GO terms in DEGs. Table S3: the list of enriched KEGG pathways in DEGs. Table S4: the expression levels of genes involving in light perception and transduction. Table S5: the expression levels of genes involving in the circadian rhythm floral induction pathway. Table S6: the expression levels and annotations of BBX genes in DEGs. [file 5572076.f1.zip › Supplemental Files/FigureS5.tif]

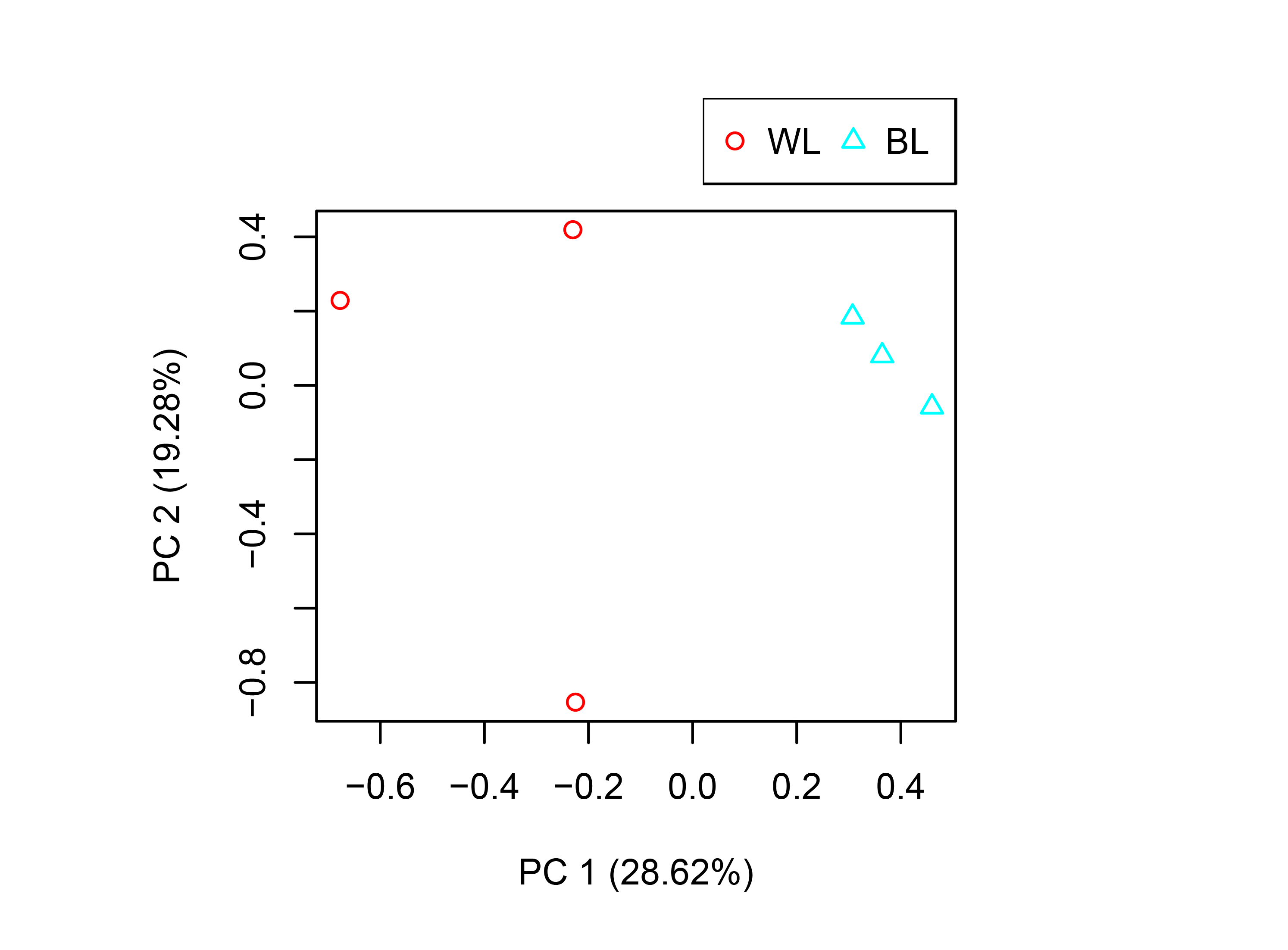

Supplement: Supplementary Materials — Figure S1: a PCA plot of samples from different treatments and replications. Figure S2: a Pearson correlation heatmap of samples from different treatments and replications. Figure S3: a pie chart illustrating the percentage of DEGs. Figure S4: a bubble chart visualizing the top 50 enriched GO terms in DEGs. Figure S5: a heatmap of gene expression involved in the light signaling pathway. Figure S6: a heatmap of gene expression which is involved in the circadian rhythm floral induction. Table S1: the expression levels and the function annotations of DEGs. Table S2: the list of top 50 enriched GO terms in DEGs. Table S3: the list of enriched KEGG pathways in DEGs. Table S4: the expression levels of genes involving in light perception and transduction. Table S5: the expression levels of genes involving in the circadian rhythm floral induction pathway. Table S6: the expression levels and annotations of BBX genes in DEGs. [file 5572076.f1.zip › Supplemental Files/FigureS1.tif]

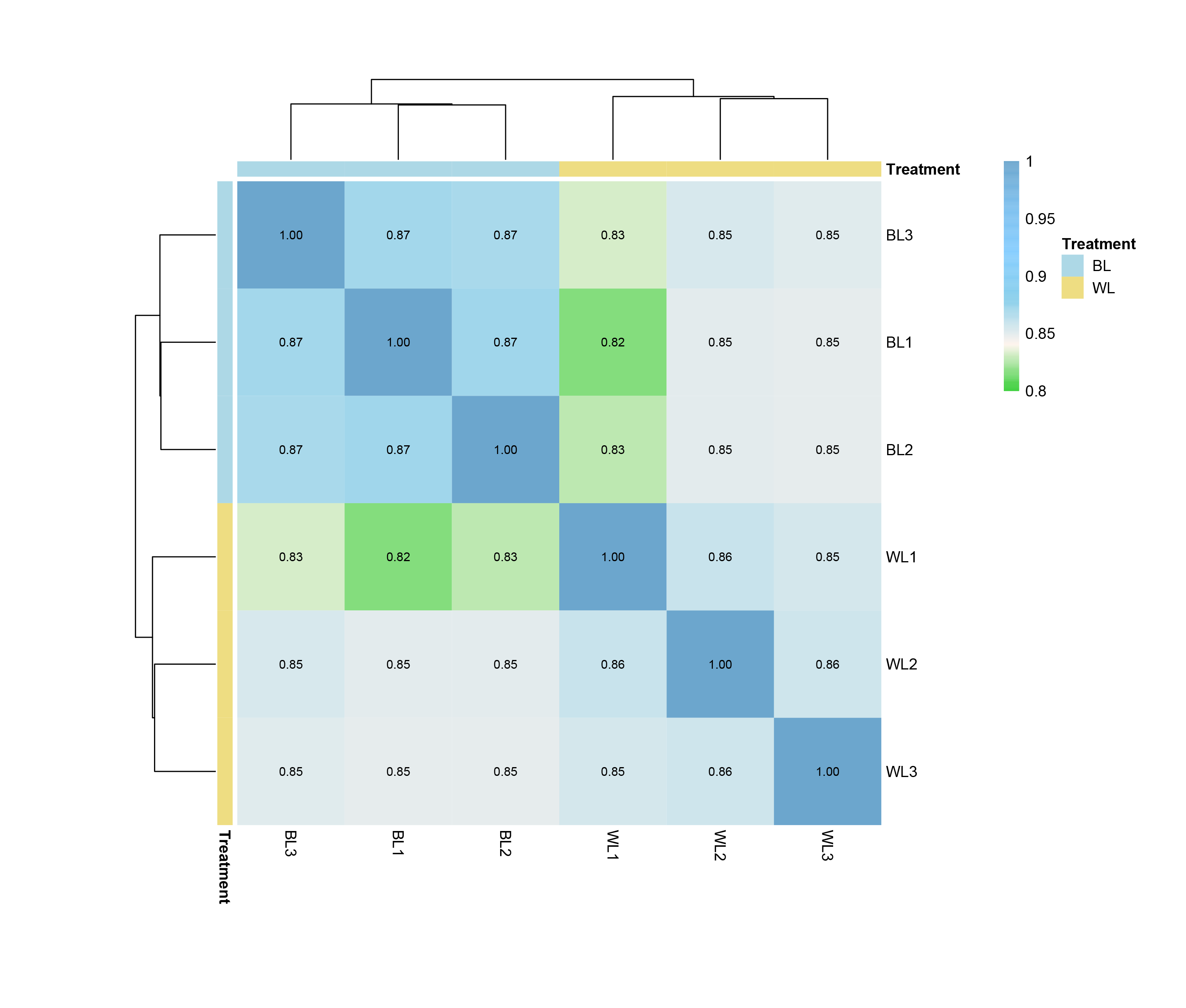

Supplement: Supplementary Materials — Figure S1: a PCA plot of samples from different treatments and replications. Figure S2: a Pearson correlation heatmap of samples from different treatments and replications. Figure S3: a pie chart illustrating the percentage of DEGs. Figure S4: a bubble chart visualizing the top 50 enriched GO terms in DEGs. Figure S5: a heatmap of gene expression involved in the light signaling pathway. Figure S6: a heatmap of gene expression which is involved in the circadian rhythm floral induction. Table S1: the expression levels and the function annotations of DEGs. Table S2: the list of top 50 enriched GO terms in DEGs. Table S3: the list of enriched KEGG pathways in DEGs. Table S4: the expression levels of genes involving in light perception and transduction. Table S5: the expression levels of genes involving in the circadian rhythm floral induction pathway. Table S6: the expression levels and annotations of BBX genes in DEGs. [file 5572076.f1.zip › Supplemental Files/FigureS2.tif]

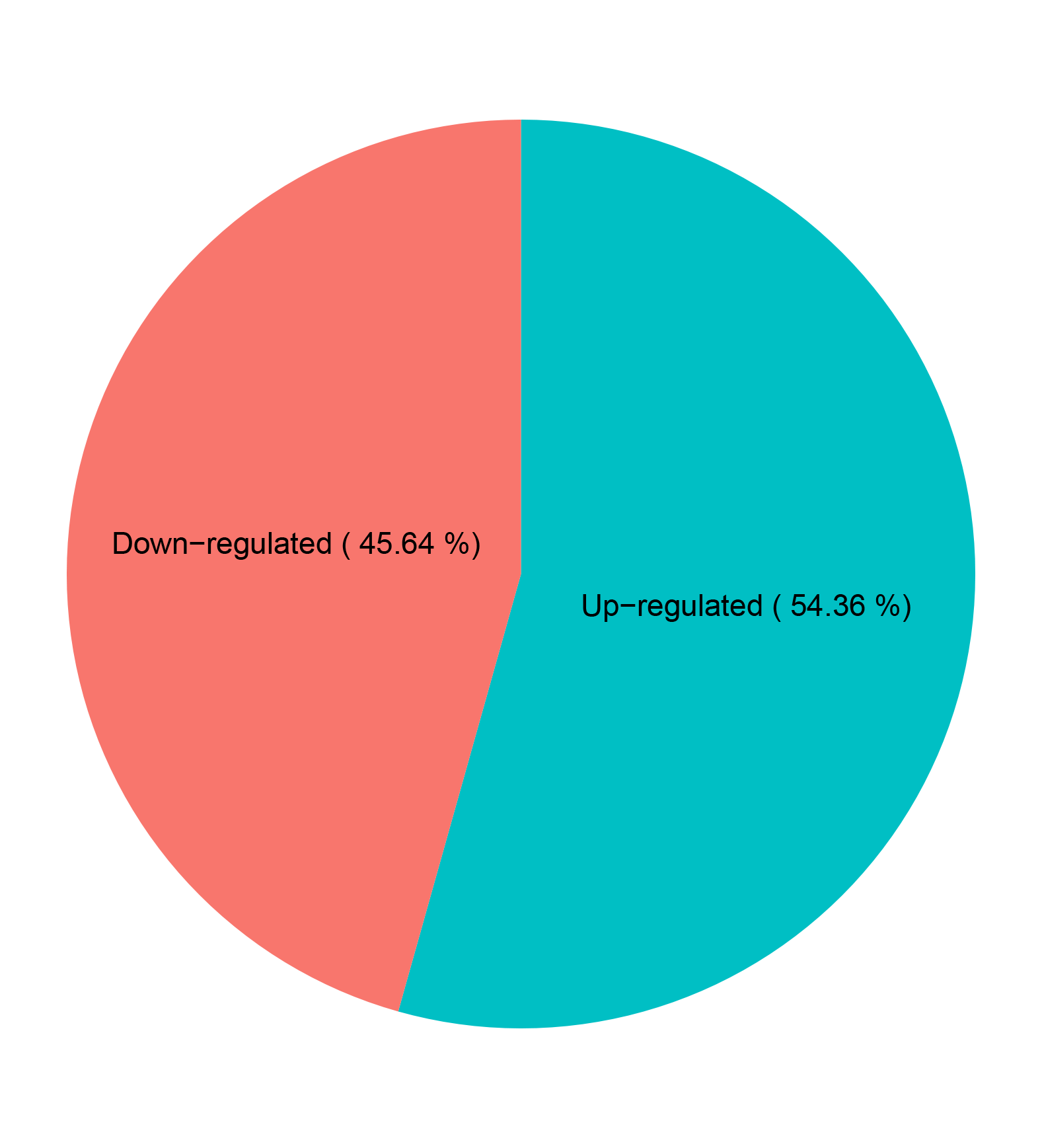

Supplement: Supplementary Materials — Figure S1: a PCA plot of samples from different treatments and replications. Figure S2: a Pearson correlation heatmap of samples from different treatments and replications. Figure S3: a pie chart illustrating the percentage of DEGs. Figure S4: a bubble chart visualizing the top 50 enriched GO terms in DEGs. Figure S5: a heatmap of gene expression involved in the light signaling pathway. Figure S6: a heatmap of gene expression which is involved in the circadian rhythm floral induction. Table S1: the expression levels and the function annotations of DEGs. Table S2: the list of top 50 enriched GO terms in DEGs. Table S3: the list of enriched KEGG pathways in DEGs. Table S4: the expression levels of genes involving in light perception and transduction. Table S5: the expression levels of genes involving in the circadian rhythm floral induction pathway. Table S6: the expression levels and annotations of BBX genes in DEGs. [file 5572076.f1.zip › Supplemental Files/FigureS3.tif]
